# Tailored recruitment interventions to improve bowel cancer screening in Arabic and Mandarin speaking groups: Modelled cost-effectiveness

**DOI:** 10.1371/journal.pone.0313058

**Published:** 2024-11-14

**Authors:** Anita Lal, Mohammadreza Mohebi, Kerryann Wyatt, Ayesha Ghosh, Kate Broun, Lan Gao, Nikki McCaffrey

**Affiliations:** 1 Deakin Health Economics, Institute for Health Transformation, Deakin University, Geelong, Australia; 2 Biostatics Unit, Faculty of Health, Deakin University, Geelong, Australia; 3 Centre for Behavioural Research in Cancer, Cancer Council Victoria, Melbourne, Australia; 4 Prevention Division, Cancer Council Victoria, Melbourne, Australia; Sichuan University, CHINA

## Abstract

**Background:**

Effective bowel cancer screening is freely available in Australia, however, there are inequities in utilisation amongst non-English speakers at home. This study estimates the health impacts and cost-effectiveness of recruitment interventions targeted at Arabic and Mandarin speaking populations in Victoria, Australia to increase bowel cancer screening participation.

**Methods:**

A Markov microsimulation model simulated the development of bowel cancer, considering National Bowel Cancer Screening Program participation rates. Culturally specific recruitment interventions e.g., community education and tailored paid media for 50–74-year-olds were compared to usual practice. A cost-utility analysis was conducted over a 50-year time horizon from a healthcare perspective, to estimate the cost per quality-adjusted life year (QALY) based on plausible effectiveness levels. Costs are in 2019 Australian dollars.

**Results:**

Intervention costs were $6.90 per person for the Arabic speaking group and $3.10 for Mandarin speakers. The estimated cost/QALY was $2,781 (95% uncertainty interval [UI]: $2,144─$3,277) when screening increased by 0.2% in the Arabic group, and an estimated 5–6 additional adenoma and cancer cases were detected. In the Mandarin group, the estimated cost/QALY was $1,024/QALY (95%UI: $749─$1,272) when screening increased by 1.1%, and an estimated 18–23 additional adenoma and cancer cases were detected.

**Conclusions:**

Culturally specific recruitment interventions to increase bowel cancer screening are inexpensive and likely to be cost-effective. Improvements in capturing language spoken at home by the National program would facilitate more precise estimates of the effectiveness and cost-effectiveness of these interventions.

## Introduction

Globally, colorectal cancer (CRC) is the third most common cancer, with almost 2 million cases diagnosed in 2020, and the second leading cause of cancer death [1]. This is despite the availability of effective screening that can reduce mortality from colorectal cancer by up to 15%-33% [2]. Countries such as Australia and England have structured bowel cancer screening programs offering free screening for eligible participants. However, disparities in the utilisation of these screening services are apparent amongst those who speak a language other than English at home [3]. These inequalities are likely to lead to differences in cancer survival and the burden borne by individuals, their families, and the healthcare system. The Victorian Government’s cancer plan 2020–24 recognises that delivering services effectively requires sensitivity to cultural and linguistic diversity [4] and one of the main aims of the Australian Cancer Plan, currently being developed by the Australian Government is to increase screening rates where disparities exist [5].

Australia has a free National Bowel Cancer Screening Program (NBCSP), which commenced in 2006 and was incrementally rolled out to select age groups. The program was fully rolled out from 2020 sending a home-based screening kit (called an immunochemical faecal occult blood test) (FOBT) every two years for eligible residents aged 50–74 years. In Australia, those who speak a language other than English at home have around 15% to 20% lower participation rates in bowel cancer screening compared to those whose main language is English (24.8%─34.3% versus 45.4%─49.2%) [6]. Similar disparities amongst ethnic groups can be found in the United Kingdom [7] and the United States [8]. Those who speak a language other than English at home are also less likely to have diagnostic follow up after a positive FOBT [6]. Arabic and Mandarin speaking communities have large steadily growing multicultural populations in Melbourne, Victoria [11] and many communities report speaking English “not well” or “not at all” [9]. Low English proficiency affects health literacy, which in turn impacts access to appropriate healthcare, including preventative health services such as cancer screening [9].

A review of effectiveness of community interventions to increase bowel cancer screening in ethnic minorities in US populations was predominately focused on Hispanic and African Americans populations limiting generalizability to other countries [10]. Four of the five counselling/community education studies demonstrated increased screening rates for the intervention group compared to controls [11–14]. Tailoring of patient education and/or messages and targeting interventions in underserved communities were common features amongst trials that increased screening [10]. In Australia predictors of screening amongst a non-Australian born study group were media exposure and health practitioners’ advice [15]. Nonetheless, there is very little evidence of the effectiveness of targeted cancer-screening recruitment interventions for culturally and linguistically diverse (CALD) groups to increase screening outside of the US that have been trialled in the community. To our knowledge the cost-effectiveness of recruitment programs for CALD populations has not been examined.

In 2019, Cancer Council Victoria was funded by the Victorian Department of Health to engage with Arabic and Mandarin speaking communities in Victoria to develop and undertake tailored recruitment interventions that complement a nation-wide bowel cancer screening mass media campaign [16]. This study aims to estimate the cost-effectiveness and long-term health impacts of interventions targeted at Arabic and Mandarin speaking populations in Victoria, Australia to increase bowel cancer screening participation. The results will help to inform whether the programs should be continued and expanded to other locations and other CALD communities. We compare the intervention to business-as-usual scenario using a cost-utility framework, over a 50-year time frame from a healthcare perspective.

## Methods

### Analytic approach

This economic evaluation uses a cost-utility analysis to compare the targeted Arabic and Mandarin recruitment intervention to ‘standard practice’ in Victoria, Australia. A cost-effectiveness model was developed in TreeAge Pro2021 using quality-adjusted life years (QALYs) as the health outcome. The costs are evaluated from a healthcare perspective as costs outside of healthcare are not considered. The costs and health outcomes are discounted at a rate of 5% as per guidelines for submissions of economic evaluations of pharmaceuticals in Australia [17] and a reference year of 2019 was used. A 50-year time horizon was utilised which covers the lifetime of participants in the program.

### Intervention description

The targeted recruitment interventions for the Arabic and Mandarin speaking community consisted of various community engagement activities and tailored paid media communications from March to September 2019. Initially, Cancer Council Victoria staff conducted interviews with community organisations, general practitioners (GPs), and prominent community ambassadors to inform the engagement strategy, the development of resources for the community education and one-on-one support. Community partnership grants were awarded to four community organisations (three Arabic and one Chinese) to develop the community education sessions. Community consultation and previous projects were used to inform which paid media and communications activities to use and which channels to utilise to target these groups most effectively. Table 1 summarises the specific interventions for each community.

**Table 1:** Arabic and Mandarin interventions 2019.

**Arabic interventions**	
**Category**	**Details of Intervention**
One to one support sessions, *Organisation 1*.	Recruitment and communication of 118 community members, one-to-one sessions with 33 community members.
Community Event, *Organisation 2*.	Community event and 10 sessions delivered to 543 people.
Bowel cancer screening information sessions, *Organisation 3*	Recruitment of 57 men and women from Arabic speaking community.
Stakeholders event	Campaign launch with Arabic community and local media, filming community champions video
GP education dinner/event	Cancer Council Victoria staff presented the importance of bowel cancer screening to GPs.
Playing cards	100 decks in Arabic
Video editing	Edited existing videos for social media and community education.
Radio ads	Arabic radio ads: 42 slots across 3 stations. Played a bonus 12 times at no extra cost
Print media ads	Half page ads in two Arabic newspapers
**Mandarin interventions**	
Community education	Delivered 19 sessions to a total of 470 community members. Shared video with their community stakeholders and conducted a radio interview to create awareness
Grant for GP education	Bowel cancer screening workshop for GPs serving the Chinese community. Cancer Council Victoria staff presented at this workshop attended by over 50 GPs (online)
Video edit	Edited existing video for social media
Radio ads	70 slots on one station. Ad received 35 bonus plays at no extra cost.
WeChat messaging	Approx. 250,000 impressions
Newspaper ad	Half page in 2 Chinese newspapers
**Both interventions**	
In language brochures and education materials, design and translation	3000
Tote bags	500
Social media production	Graphic design
Facebook Video ads	Targeted Facebook ads for 3 months

#### Arabic community

The term ‘Arabic speaking’ represents people from 22 countries. With such a varied community, community engagement activities were focused on communities with the largest populations in the targeted age range, of Lebanese, Iraqi and Egyptian communities [22]. One-on-one interviews were conducted with four community organisations, three GPs and seven prominent community leaders to inform development of the interventions. These community consultations explored the barriers to participation in the NBCSP and emphasized the role of humour in engaging the community, as well as the value of using community champions to discuss sensitive issues like bowel cancer screening.

The education sessions were run by grassroots community organizations who provide services for established migrants and newly arrived refugees. This included individual support sessions for 33 community members, educational events reaching over 543 people at Arabic community organisations, and in-language radio ads, Facebook videos, and newspaper ads. Special resources such as playing cards and tote bags with bowel cancer screening messages were also distributed. The in-language 30 second video advertisements on Facebook went to 65,000 users, a 15 second YouTube ad had 53,000 views and a 45 second skippable version with around 20,000 views (completion rate of 35%).

#### Mandarin community

For the Mandarin-speaking community, 19 education sessions were held, reaching 470 people. The tailored media included 70 radio slots, a WeChat campaign with 250,000 impressions, and in-language newspaper ads. The interventions also featured online educational workshops for 50 GPs and online outreach, including targeted Facebook video ads.. One-on-one interviews were conducted with five community organisation staff who provides support services to those affected by cancer and ten community ambassadors from the Mandarin community. The interviews examined the barriers faced by the community in participating in the NBCSP and provided recommendations for increasing participation among the Mandarin-speaking community.

### Comparator

The comparator is a no tailored intervention scenario, i.e. an FOBT kit is sent to participants with a standard letter and an information brochure in English. The back of the brochure has links to 22 language translations.

### Eligible population

The eligible population consisted of Arabic and Mandarin speaking adults aged 50–74 in 2019 residing in Victoria. The population numbers are based on estimates from Australian Bureau of Statistics (ABS) Census data in 2016 and 2021 who reported language spoken at home as Arabic and Mandarin for year of age from 50 to 74 years in Victoria [18, 19]. Linear interpolation was used to estimate population numbers in 2019. The population data used can be found in S1 Table. We estimated 17,522 individuals spoke Arabic and 38,660 spoke Mandarin at home in the eligible age group in 2019 and resided in Victoria.

### Intervention effectiveness

Calculation of participation rates in the NBCSP requires the number of screening invitations sent out (the denominator) as well as the number of completed screening kits returned (the numerator). The percentage of kits returned reporting ‘unknown’ language spoken at home was 83% in 2019 and 82% in 2018 based on data provided by the Australian Centre for the Prevention of Cervical Cancer (ACPCC) from the National Cancer Screening Register [20] (accessed 9/9/2022). With such low percentages of recorded language spoken, and data not recorded for the number of kits sent out to Arabic and Mandarin speaking communities, we explored using proxy areas. We examined local government areas in Melbourne, Victoria with the largest proportions reporting low English language proficiency and the highest concentration of Arabic and Mandarin speakers [9]. The analysis was narrowed down to the areas with the highest percentage of people speaking Arabic and Mandarin at home in the target age group of 50–74 years from Census 2021 data, being Hume with 22% of the Arabic population and Whitehorse with 29% of the Mandarin population in Victoria [18, 19]. These areas were also included in the Victorian tailored paid media advertising, as well as the larger national campaign, which is separate from the language-specific community interventions. As the areas included residents speaking English and other languages, changes in screening rates for the whole of Victoria were examined, as well as South Australia (SA) as a comparison state where the tailored interventions did not take place, but the national media campaign also occurred. The national campaign included in language advertising targeting Arabic, Mandarin, Cantonese, Greek and Italian speakers aged 50–74 with print, radio, digital and out of home advertising.

Engagement activities ran from March to July 2019 and tailored paid media ran from July to September 2019. To calculate the effectiveness of the intervention, participation in 2019 when the targeted recruitment interventions occurred for Arabic and Mandarin speaking groups was compared with 2018 when there were no targeted recruitment interventions for the Arabic or Mandarin community. It is unlikely that there were significant changes in the population composition between the years under examination. In 2020 participation was affected by COVID-19 and was therefore not an appropriate comparison year.

#### Statistical analysis

We used negative binomial regression to explore the statistical significance of the number of screening kits returned. To enable estimation of the effects of the intervention on return rates among those sent a NBCSP FOBT kit we used a negative binomial regression to model monthly return kits in the intervention and control periods in an interrupted time-series design setting, as used in similar studies of impact on bowel cancer screening [21]. As the engagement activities ran from March to September 2019, the intervention timepoints used were March to November in 2018 and 2019. We used two months after the intervention ended to allow for a time lag to participate as per previous studies [22] The nominator of the rates (i.e. independent variable) was the monthly number of return kits, and the number of people sent an FOBT kit in each month was used as an offset term in the model in order to estimate monthly rates. Incidence rate ratio of the intervention period versus the control period and the 95% confidence intervals were estimated. A Huber-White robust estimator with an autocorrelation structure was used to account for monthly serial correlation [23]. All statistical analyses were performed with STATA 17 [24].

To estimate the participation rates of Arabic and Mandarin speaking participants, the number of screening invitations sent out to Arabic and Mandarin speaking participants was approximated based on the study population estimates described previously in the ‘Eligible population’ section. Using customised data from the ACPCC [20] for the number of kits returned, the participation rates in the baseline year (control period) were estimated.

We tested different effect sizes (increases in screening participation) to determine the minimum increase that results in a cost-effective intervention as well as a plausible “best case” increase in screening participation scenarios. We assumed that the intervention effect lasted for 1 year and colonoscopy rates increased to English speaking rates for 1 year.

### Ethics approval and consent to participate

The study was approved by Deakin University Human Ethics Advisory Group HEAG-H 96_2021. All data are non-identifiable therefore the need for informed consent was waived by the Deakin University Human Ethics Advisory group. All methods were carried out in accordance with the National Statement on Ethical Conduct in Human Research 2007.

### Model structure

#### Overview

**Fig 1:**
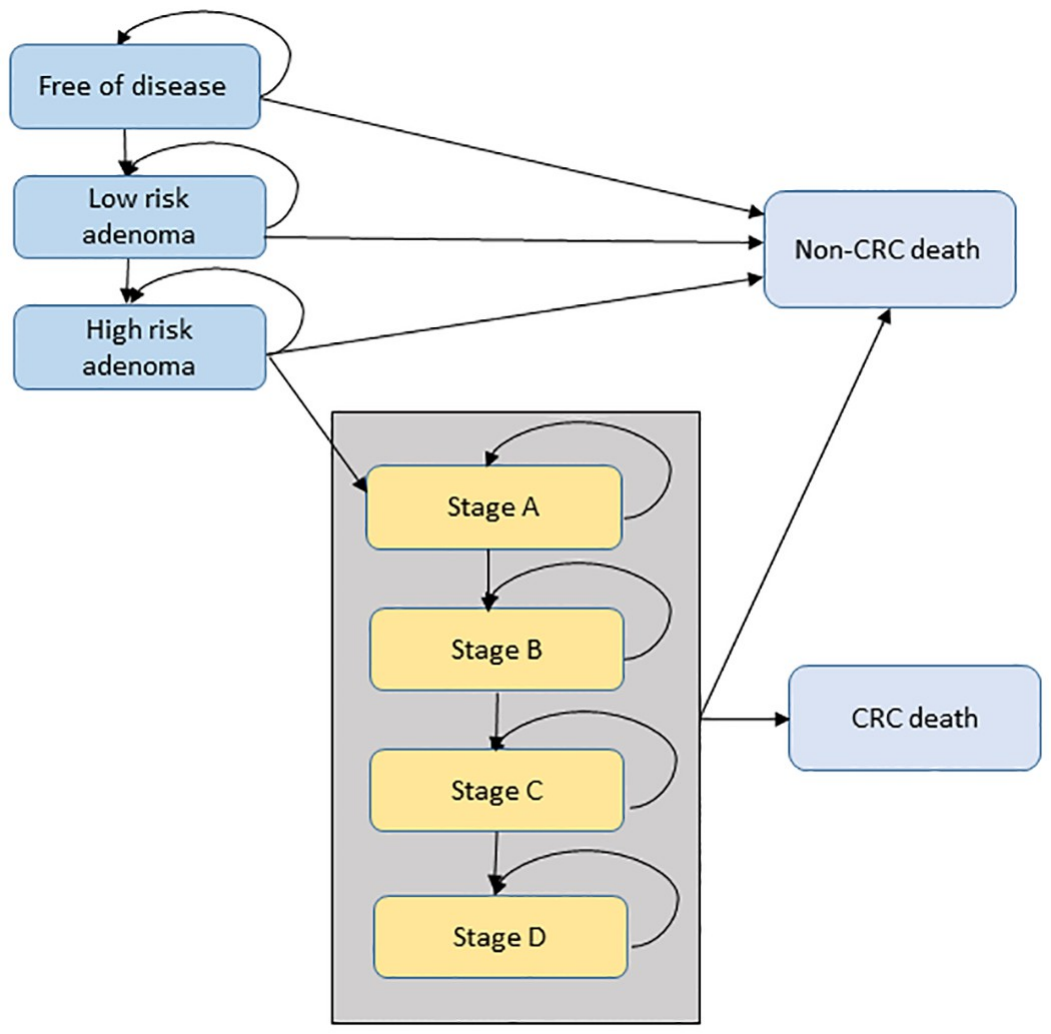
PRISM-CRC model Markov health states. Notes: CRC: colorectal cancer.

The Priority Population Microsimulation CRC (PRISM-CRC) model [25] was used to estimate the cost per QALY gained and the lifetime health outcomes for 10,000 individuals for the intervention when compared to usual practice.. The model simulates the development of CRC considering participation rates in the NBCSP and estimates the health outcomes—cases of CRC and deaths for the intervention compared to a business-as-usual scenario. The main difference from participation in the NBCSP versus non-participation is the stage when CRC is diagnosed. The simulated cohort moves through the Markov model in 1-year cycles until death [26]. (Fig 1) At the end of each 1-year period the individual person either moves to a different health state or stays in the current health state. The model consists of three parts 1) natural cancer development 2) screening and 3) colonoscopy surveillance. Participants enter the model in either screening or natural cancer development.

#### Natural development of CRC

It is assumed that colorectal cancer develops through the adenoma-carcinoma pathway. The natural development of the CRC component of the model influences the movement of patients to different health states. The seven health states within the model are free of disease, low risk adenoma, high risk adenoma and the four stages of CRC classified according to Australian Clinico-Pathological Staging (ACPS) system from A to D, indicating how far the cancer has spread anatomically [27]. Other colorectal cancer models have used a similar structure [28]. A non-symptomatic cancer can remain undiagnosed or may be symptomatically diagnosed. If the cancer remains undiagnosed, it can progress from Stage A through each stage, to Stage D. Survival after diagnosis depends on the cancer stage at diagnosis and the time since diagnosis. The model assumes i) that the underlying risk of CRC remains constant using CRC incidence rates in 2019, ii) patients who survive more than five years after diagnosis are assumed to be cancer survivors and have general population mortality rates with no recurrent CRC and iii) a maximum age of 100 years. Increased risk of CRC due to family history is not included.

#### CRC screening

**Fig 2:**
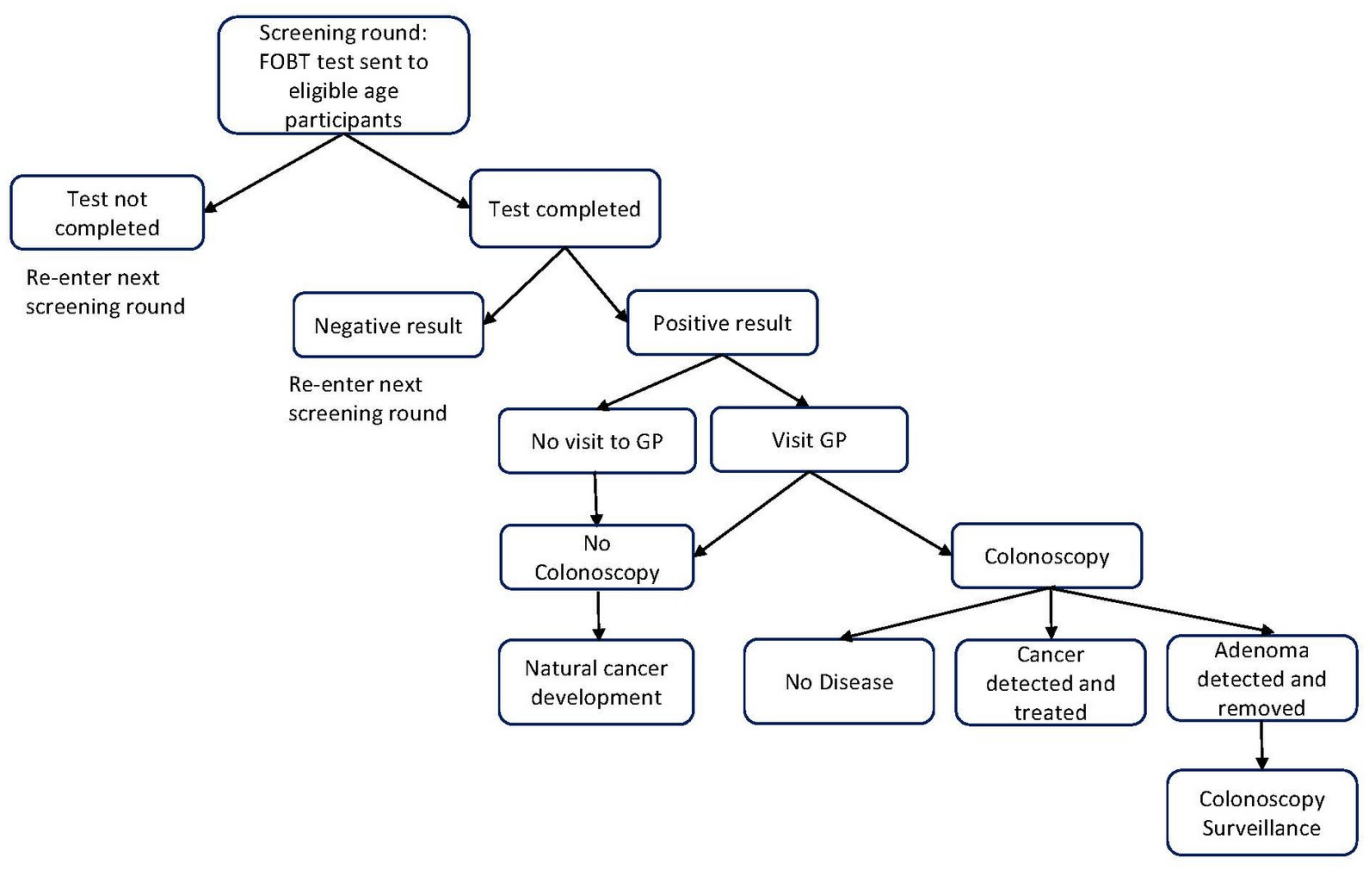
Screening and health outcomes pathway.

The model simulates the patient pathway in 2019 when screening kits were sent out to people aged 50, 52, 54, 56, 58, 60, 62, 64, 66, 68, 70, 72 and 74 years by the NBCSP (Fig 2). FOBT kits are assumed to be sent to all eligible participants to complete at home. Tests that were returned were assumed to be completed correctly. An individual returning a negative test would re-enter the screening program and be sent an FOBT kit when they were eligible again. An individual returning a positive test would visit a GP for follow up, usually resulting in a referral for a colonoscopy procedure. The three possible outcomes following colonoscopy are adenoma detected, cancer detected or no disease. It was assumed that no adverse events occurred following a colonoscopy. If the individual decides not to have a colonoscopy, they move to the natural cancer development part of the model and if cancer is present, it would be detected via symptoms.

#### Colonoscopy surveillance

If an adenoma is detected via colonoscopy, the individual is moved to the colonoscopy surveillance section of the model. Individuals visit a GP every five years to obtain a referral for the colonoscopy. Adenomas detected are removed. Colonoscopy surveillance is assumed to take place until age 75 years. It was assumed that no adverse events occurred following a colonoscopy.

A summary of the key input parameters used by the PRISM-CRC model is provided in Table 2. The model was built in TreeAge Pro software [29]. Survival by stage 1–5 years is detailed in S2 Table.

**Table 2:** Key model parameters used by the Priority Population Microsimulation Colorectal Cancer (PRISM-CRC) model.

Model Parameter	Value (uncertainty range)		Source & notes
Colonoscopy assessment rate, 2019			
Language other than English	54.3%		Australia 2019, AIHW 2021 [30]
English	63.1%		
Screening positivity rate, 2019			
Language other than English	6.4%		Australia 2019, AIHW 2021 [30]
English	6.9%		
**2019 population incidence rate CRC**			
50–54 years	0.0007		Calculated from age-specific rate 100,000, Victorian Cancer Registry [31]
55–59 years	0.0008		
60–64 years	0.0012		
65–69 years	0.0016		
70–74 years	0.0019		
75–79 years	0.0026		
80–84 years	0.0031		
85+ years	0.0037		
**Population Incidence of progressive adenomas, age adjusted**			
50–54	0.0038		Calculated as 20 years prior to CRC incidence [28, 32] ie age 50–54 is incidence of cancer age 70–74.
55–59	0.0041		
60–64	0.0062		
65+	0.0068		
**Incidence of all adenomas**			
50–54	0.0012		Incidence of adenoma adjusted for proportion of all adenomas that develop into progressive adenomas of 24% [33]
55–59	0.0015		
60–64	0.0019		
65+	0.0025		
**Incidence per person, low risk adenomas**			
50–54	0.0160		Incidence of all adenomas minus incidence of progressive adenomas (from above)
55–59	0.0173		
60–64	0.0257		
65+	0.0282		
**Transition probability**			
Low risk to high risk	0.02 (0.01–0.04)		Frazier et al [34]
High risk to CRC	0.05 (0.02–0.10)		
Stage 1 to Stage 2	0.05 (0.02–0.10		
Stage 2 to Stage 3	0.28 (0.10–0.50)		
Low risk to high risk	0.02 (0.01–0.04)		
**Disease prevalence from colonoscopy assessment**			
Free of disease	87.40%		Australia, AIHW 2021 [30]
Polyps or Low risk adenoma	6.09%		
High risk adenoma	5.65%		
CRC present	3.13%		
**Distribution of CRC by stage and screening status**	**Screened**	**Not Screened**	
Stage A	49%	22%	Victorian Cancer Registry dataset 2009–2019* (accessed 22/3/2023 and 14/6/2023)
Stage B	19%	32%	
Stage C	24%	27%	
Stage D	8%	19%	
**Probability of CRC diagnosis if not screened**	**Diagnosis probability**	**Range**	Calculations based on distribution of CRC by stage, not screened (from cells above) [35]
Stage A	0.22	0.20–0.24	
Stage B	0.35	0.25–0.45	
Stage C	0.59	0.49–0.69	
Stage D	1.000	0.90–1	

Notes:

* Victorian Cancer Registry dataset under license for this study. AIHW; Australian Institute for Health and Welfare, CRC; colorectal cancer

### Cost effectiveness outcomes

#### Cost utility analysis

Cost-utility analysis (CUA) compares the costs and quality adjusted life years (QALYs) of two different courses of action. QALYs were estimated by multiplying the utility score associated with a health state (represents quality of life) by the length of life lived in that health state (represents quantity of life) [36, 37]. In this study utility scores based on a meta-analysis by CRC stage were used, with stages A to C valued at 0.74 and stage D valued at 0.68 [38]. For the population without CRC, Australian population norm utility scores were valued at 0.80 for ages 50–69, 0.76 for ages 70–79 and 0.70 for 80 years and over [39]. Cost-effectiveness outcomes for the CUA are reported as an incremental cost effectiveness ratio (ICER) which is calculated as the difference in costs between the intervention and the control group, divided by the difference in QALYs. A willingness-to-pay threshold of A$50,000 per QALY gained was used to decide if the intervention would be considered cost-effective [40, 41]. The cost-effectiveness model designated 2019 as the reference year, with a discount rate of 5% per year applied to all costs and health outcomes. All costs are expressed in 2019 Australian dollars (A$) and converted to 2019 prices using the AIHW total health price index [42].

### Cost analysis

#### Intervention costs

Costs for the targeted recruitment programs were obtained from Cancer Council. (S3 Table). The costs included the community grants, education materials, promotional items, media production and airing of advertisements and Cancer Council Victoria staff time. The NBCSP costs include the FOBT kit, return postage and laboratory processing for Medicare Benefits schedule (MBS) item 66767 [43]. If a patient needed a colonoscopy, they are assumed to be referred to a GP for a consultation costed as MBS item 23 [43]. The cost of colonoscopy procedure (following a positive FOBT) was assigned based on whether a polypectomy was performed and whether complications occurred.

#### Cost savings

Healthcare cost savings from the CALD targeted recruitment interventions were estimated by multiplying the number of cases of CRC averted by the treatment costs. Treatment costs were based on CRC stage taken from an Australian study that estimated the cost of cancer therapies [44]. Half the patients in Stage D receiving chemotherapy also received bevacizumab medication at a total cost of approximately $12,375, therefore an average of the cost of treatment with and without bevacizumab was used. The dosage and duration for bevacizumab was taken from Ananda et al to be weekly dose of 200mg over 28 weeks [44]. Costs are detailed in Table 3.

**Table 3:** Healthcare costs of National Bowel Cancer Screening Program and treatment costs.

Resource	Value (range)	Source & notes
**FOBT costs**		
FOBT kit	$8.00 (10–12)	Assumption based on previous Australian study [28]
Packaging and postage	$2.40	Mailing costs and return postage [45, 46]
Test analysis in laboratory	$17.85	MBS item 66767, Department of Health [47]
**Colonoscopy**		
General Practitioner consultation	$37.60	MBS item 237, Department of Health [47]
Colonoscopy, no biopsy (without complication)	$2,258	AR-DRG item G48B, Independent Hospital Pricing Authority [48] inflated to 2019
Cost of colonoscopy with polypectomy	$4,203	AR-DRG item G47B, Independent Hospital Pricing Authority [48] inflated to 2019
**CRC Treatment**		
Stage A	$40,999 ($37,657 to $46,025)	Mean costs inflated to 2019 dollars, Ananda et al [44] (plus or minus 10%)
Stage B	$52,594 ($48,307 to $59,041)	
Stage C	$95,754 ($87,948 to $107,492)	
Stage D	$90,272 ($82,913 to $101,338)	

Notes: FOBT: faecal occult blood test; MBS: Medicare Benefits Schedule; AR-DRG: Australian Refined Diagnostic related group. Victorian Cancer Registry Dataset was used under license for the current study. Costs inflated to 2019 dollars using AIHW health price index

### Uncertainty and sensitivity analyses

Uncertainty analyses were undertaken to evaluate how robust the results were to the assumptions made in the analysis. A probabilistic multivariate uncertainty analysis was conducted using Monte Carlo simulation method. Means and 95% uncertainty intervals for incremental costs, are reported based on 2,000 trials of 10,000 patients each, using TreeAge software. Uncertainty parameters are presented in Table 2. For Mandarin speaking groups we tested 1) a 0.09 increase in screening and 2) 2.4% increase in screening. For Arabic groups we tested 1) a 0.02% increase and a 2) 1.3% increase in screening. We conducted sensitivity analyses (SA) to evaluate the impact of using fixed discount rates of 3.5%, and 0% per year as per guidelines for submissions of economic evaluations of pharmaceuticals in Australia [17]. We tested the model validity alongside the number of bowel cancer cases in 2019 from the Victorian Cancer Registry [31].

## Results

### Effectiveness

Based on the data provided by the ACPCC for the number of kits returned and the Mandarin and Arabic study population estimates, the NBCSP participation rates in the baseline year (control period) were estimated at 30.7% for Arabic speaking and 40% for Mandarin speaking.

The calculated incidence rate ratios and reported participation rates for the Mandarin and Arabic proxy areas and the two states are reported in Table 4. All three locations in Victoria had a significant increase in the rate of screening of 1.20 (Whitehorse), 1.17 (Hume) and 1.18 (Victoria) in 2019 compared to 2018. In SA, the rate of screening was 1.5 times the rate compared to 2018 but was not significant. In SA where there were no additional non-English interventions, reported annual screening increased by 4.3% [26]. The increase in SA is assumed to represent the impact of the national media campaign in 2019. The incremental effect size for Whitehorse was 1.1% when compared to SA. For Hume there was no increase when compared to SA.

**Table 4:** Incidence rate ratios and participation rates.

	Whitehorse (Mandarin poxy)	Hume (Arabic proxy)	Victoria	South Australia
Reported Participation rates 2018	47.8%	40.1%	44.2%	46.9%
Reported participation rates 2019	53.2%	44.4%	49.0%	51.2%
Increase in participation	5.4%	4.3%	4.8%	4.3%
Incremental increase compared to SA	1.1%	0	0.5%	NA
Incidence rate ratio (95% CI)	1.20 (1.05 to 1.37) p<0.01	1.17 (1.03 to 1.33) p<0.05	1.18 (1.05 to 1.33) p<0.01	1.15 (0.98 to 1.33), p = 0.08

Notes: The incident rate ratios are from negative binomial distribution analyses. Participation rates calculated for Local Government Areas were sourced from Australian Centre for the Prevention of Cervical Cancer based on data provided by National Cancer Screening Register. State data is from Australian Institute for Health and Welfare [49]; CI: Confidence Intervals, NA: not applicable.

### Cost-effectiveness findings

The following results assume the culturally specific recruitment interventions ran for 1 year, with the effect lasting 1 year and returning to baseline in year 2.

#### Arabic program

Total costs of implementing the targeted bowel cancer screening program for the Arabic speaking population was $120,863 for 1 year. The costs of community education programs were $18.00 per participant and $1.10 per person for tailored media. The average overall cost was $6.90 per person aged 50 to 74 years. Using the difference in the incident rate ratios for SA and Hume to get a minimum increase in the rate of screening of 0.2%, the estimated ICER was $2768 per QALY (95% uncertainty interval [UI]: $2144 to $3277) and an estimated 4–6 additional cases of adenoma or cancer were detected when compared to usual practice (Table 5). If an increase in screening of 1.3%.

**Table 5:** Summary of results for targeted Arabic and Mandarin bowel cancer screening recruitment interventions.

**Arabic Program**			
	**Base case** **(no targeted program)**	**0.2% increase in screening, 1 year**	**1.3% increase in screening, 1 year**
Incremental costs		$14.22	$13.19
Incremental QALYs		0.005	0.018
Mean QALYs gained per person	11.047 (11.037 to 11.053)	11.052 (11.043 to 11.058)	11.064 (11.056 to 11.070)
Mean health-care costs per person	$836 ($637 to $1,056)	$851 ($655 to $1,068)	$850 ($651 to $1,070)
Incremental cost-effectiveness ratio*	─	$2768 ($2144 to $3277)	$749 ($569 to $902)
Mean cases of adenoma and cancer detected	38 (26 to 50)	43 (30 to 56)	44 (31 to 57)
**Mandarin Program**			
	**Base case** **(no targeted program)**	**1.1% increase in screening, 1 year**	**2.4% increase in screening, 1 year**
Incremental costs		$9.94	$20.94
Incremental QALYs		0.010	0.024
Mean QALYs gained per person	11.229 (11.221 to 11.234)	11.239 (11.230 to 11.245)	11.253 (11.244 to 11.259)
Mean health-care costs per person	$724 ($514 to $971)	$734 ($525 to $979)	$745 ($539 to $989)
Incremental cost-effectiveness ratio*	─	$1020 ($749 to $1,272)	$884 ($714 to $1,115)
Mean cases of adenoma and cancer detected	131 (109 to 153)	149 (125 to 173)	154 (130 to 178)

Notes: 95% Uncertainty intervals in brackets are calculated by re-running the model 2,000 times with 10,000 participants while repeatedly Monte Carlo sampling with replacement from the distributions of input parameters to estimate uncertainty in the outcome metrics.

*Difference in cost divided by change in QALYs, compared with the base case. Costs are given in A$2019.

Occurred, (3% less than the reported participation rate), the estimated ICER is $749 (95% UI: $569 to $902) and an estimated additional 5–7 adenoma or cancer cases were detected. Both scenarios have 100% of uncertainty iterations below $50,000, the threshold considered cost-effective for Australia [40, 41]. Mean healthcare costs were similar in the two scenarios which may reflect the short duration of the intervention.

#### Mandarin program

Total cost of the targeted Mandarin bowel cancer screening program was $120,337 for 1 year. The costs of the community education programs were $16.80 per participant and $0.94 per person for tailored media. The overall costs were $3.10 per person aged 50 to 74 years. If the Mandarin program resulted in an increase of 1.1% in bowel cancer screening, (the incremental increase above South Australia) the estimated ICER is $1,020 per QALY (95% UI: $749 to $1272) when compared to usual practice and 16–20 additional cases of adenoma and cancer detected. If the program resulted in an increase in screening of 2.4% (3% less than the reported increase in screening) the estimated ICER is $884 (95%UI: $714 to $1115) and approximately 21–25 more cases of adenoma and cancer detected.

#### Model validation

The Victorian Cancer Registry reported 1989 bowel cancer cases in Victoria for those aged 50–74 in 2019 [32]. Based on Census data, the Mandarin speaking population aged 50–74 represents 2.3% of the Victorian population (Table 5). For the Mandarin cohort the model predicts 2,323 total bowel cancer cases over 50 years, i.e. 47 per cases year which is consistent with 2.3% of reported bowel cancer cases in 2019 (46 cases). The Victorian Cancer Registry reported the crude rate of deaths from bowel cancer in 2019 to be 17.2 to 21.1 per 100,000 for males and females respectively [32]. This equates to 7 to 8 deaths in a population of 38,660 per year. Our model estimates 409 deaths over 50 years or an average of 8 deaths per year.

#### Sensitivity analyses

When varying the discount rates to 0% and 3.5% per year the recruitment interventions remained very cost-effective. At the lowest level of increase in participation (0.2%) for the Arabic speaking group and no discount on costs or benefits, the estimated ICER was $2,152 (95% UI: $1,241 to $2,514). For the Mandarin speaking groups at the lowest level of increase in participation (0.9%) the estimated ICER was $755 (95% UI: $78 to $1,738). Full details are in S4 Table.

## Discussion

In our study we estimate that very small increases in bowel cancer screening by the Arabic and Mandarin speaking groups from the targeted culturally specific recruitment interventions for CALD communities in Victoria would be cost-effective. We used very conservative ranges of increases which still resulted in very cost-effective ICERs less than $3000. The program only improved the QALY marginally due to the relatively low incidence of CRC with only a small percentage of screened people diagnosed with cancer over the 1-year period. The interventions were inexpensive to run at $120,000 per year and given that treatments costs are on average $40,000 to $90,000 per patient, if the program prevented at least 2 or 3 cases of bowel cancer the value for money is evident. This is the first study to analyse the effectiveness and cost-effectiveness of targeted CALD recruitment interventions to increase bowel cancer screening and potentially they are value for money.

Comparison with other studies is difficult given the lack of similar studies. Our estimates of value for money are below the $3,000/QALY and considering broader benefits such as cost savings in productivity losses due to absenteeism and premature death, would likely make the results even more favourable. For context Australia’s national bowel screening program was estimated at $3014 per life year saved at the 40% participation rate in 2015 [28]. In a review of community interventions to increase screening in ethnic minorities in the US populations, 4 of 5 (80%) counselling/community education studies, demonstrated increased screening rates for the intervention group compared to controls [13]. However, the interventions analysed included patient mailings, electronic/multimedia and telephone outreach that were not similar in nature to the recruitment interventions analysed in our study. A factor that improved screening rates that was common to our study was targeting interventions in underserved communities where the benefits are potentially the highest.

At current participation rates the NBCSP is underutilised and substantial additional investment could be spent on improving participation while still remaining cost-effective [50]. The costs of the intervention in our study ranged from $3─$7 per person. The estimated maximum spending levels of effective interventions to improve screening rates from 40% to 60%, has been estimated to be AUD$214–$502 per person [50]. Given that a main aim of government screening strategies in Australia is improving equity of access for disadvantaged groups [51], the expansion of these types of interventions for other languages indicates the potential benefits of scaling up similar recruitment initiatives to a state or national level.

As part of the wider evaluation, interviews with Arabic and Mandarin community organisation staff were conducted a few months after their sessions to assess the feasibility of the recruitment interventions [16]. Several strengths of the interventions should be noted. The paid media intervention was able to leverage from a national mass media campaign to develop and create localised in-language communication assets that resonated with the Victorian community (eg, a video with a local Mandarin-speaking GP and a video with trusted Arabic speaking community leaders). The grants scheme model allowed community organisations autonomy over the organisation of events, and this helped them build mutually beneficial relationships with their communities. The written in-language resources were able to be shared amongst family and friends and enable staff to circulate them to new clients continuously. This aspect, as well as video resources being shared on social media are an important sustainable part of the program. Being able to host sessions in different Arabic dialects allowed a sense of trust amongst attendees. However, finding a balance between providing in-language facilitators and cancer expertise was a challenge. Bi-lingual educators allow for a rapport to be built but they lack in-depth knowledge of bowel cancer. A Cancer Council Victoria facilitator with a translator present did not easily facilitate open conversations with attendees. Community organisations from both languages highlighted that deep-rooted cultural beliefs were a significant barrier to bowel cancer screening and continued education will be important [16]. The differences in uptake of screening for the two groups may reflect the variability in the levels of health literacy that need to be addressed. The feedback from the Mandarin community was that they had attended previous sessions and their knowledge was already high [16].

There are some caveats around the measurement of the effectiveness of the interventions and the results should be viewed as possible scenarios. It is difficult to determine whether the increases in participation are due to the additional state-based intervention in Victoria and how much is due to the national campaign. However, having South Australia as a control state was the best way to addresses this. When making screening participation comparisons in 2019 and 2018, it should be noted that 2019 was the final year of the phased in program with the last age groups added (52- and 56-year-olds) and in 2018 two age groups were added (62- and 66-year-olds) to the NBCSP [52]. Recruitment campaigns can also prompt testing outside of the NBCSP for example they may purchase a kit from the chemist, speak to their doctor about having a colonoscopy etc. This study only measures effects within the NBCSP. Therefore, the participation rates used in the modelled analysis are most likely underestimated. The data quality of recorded FOBT kits by language spoken at home placed large limitations on the accuracy of the effectiveness analysis. Encouragingly, in 2020 the percentage of kits returned with language unknown or not recorded had reduced to less than 20%, however, uncertainty remains around the language spoken of the invitees [20]. Continuous improvement is needed such as improved data linkage ideally with Medicare to capture language spoken at home. As with all modelling studies there are limitations. The model contains several assumptions: we assumed that patients who did not have a follow-up colonoscopy after a positive FOBT shifted to the not screened arm; the reported diagnostic results were used to calculate the probabilities of adenoma, cancer and no disease in the model [53] rather than the sensitivity and specificity of FOBTs and colonoscopies and the model assumes the incidence of CRC remains stable over the 50-year period.

## Conclusions

Targeted culturally specific recruitment interventions to increase bowel cancer screening in Arabic and Mandarin speaking groups have the potential to provide value for money. Improvements in data capture of language spoken at home by the NBCSP will ensure that interventions can be evaluated accurately for their effectiveness and cost-effectiveness. Ideally this would lead to improvements in the use of resources to address inequalities of cancer screening in the community. The ultimate benefit will be better cancer-related outcomes amongst culturally and linguistically diverse groups, leading to better health, higher rates of long-term survival and reductions in the social and economic burden borne by themselves, their families, the healthcare system, and the community.

## Supporting information

S1 Table Estimated population Arabic and Mandarin speaking groups age 50–74 years, Victoria 2019. (PDF)

S2 Table Survival by stage 1–5 years, screened and not screened. (PDF)

S3 Table Costs of Arabic and Mandarin program 2019. (PDF)

S4 Table Results of varying discount rates. (PDF)
