# Supplementary material for: Tailored recruitment interventions to improve bowel cancer screening in Arabic and Mandarin speaking groups: Modelled cost-effectiveness
Source: PLoS One. 2024 Nov 14;19(11):e0313058. doi: 10.1371/journal.pone.0313058 (PMC11563420; doi:10.1371/journal.pone.0313058)
Supplement: S1 Table — (PDF) [file pone.0313058.s001.pdf]

S1 Table: Estimated population Arabic and Mandarin speaking groups age 50-74 years, Victoria 2019.

	Estimated population numbers	
Age (years)	Arabic	Mandarin
50	1059	2142
51	999	2108
52	1006	2253
53	928	2260
54	905	1668
55	919	1759
56	915	1818
57	871	1942
58	821	2311
59	766	1840
60	757	1767
61	722	1711
62	703	1535
63	670	1712
64	645	1726
65	613	1690
66	589	1551
67	544	1342
68	547	1082
69	489	942
70	453	897
71	446	793
72	400	696
73	403	593
74	352	523
Total	17,522	38,660

Notes: Estimated based on linear interpolation of 2016 and 2021 Census data from the Australian Bureau of statistics [1]

1. Language spoken at home by single year of age [Census TableBuilder] 2016 and 2021, accessed 1 July 2022.
