# Supplementary material for: Tailored recruitment interventions to improve bowel cancer screening in Arabic and Mandarin speaking groups: Modelled cost-effectiveness
Source: PLoS One. 2024 Nov 14;19(11):e0313058. doi: 10.1371/journal.pone.0313058 (PMC11563420; doi:10.1371/journal.pone.0313058)
Supplement: S2 Table — (PDF) [file pone.0313058.s002.pdf]

S2 Table: Colorectal cancer survival by stage 1-5 years, screened and not screened.

Survival by stage 1-5 years, screened					
**Stage**	**Year 1 (95% CI)**	**Year 2 (95% CI)**	**Year 3 (95% CI)**	**Year 4 (95% CI)**	**Year 5 (95% CI)**
Stage 1	0.991 (0.939, 0.999)	0.983 (0.932, 0.996)	0.983 (0.932,0.996)	0.938 (0.873,0.970)	0.917 (0.845,0.956
Stage 2	1.000 (1.000,1.00)	0.977 (0.849, 0.997)	0.977 (0.849,0.997)	0.977 (0.849,0.997)	0.977 (0.849,0.997)
Stage 3	0.981 (0.874, 0.997)	0.981 (0.874, 0.997)	0.943 (0.835, 0.981)	0.925 (0.811, 0.971)	0.901 (0.778,0.958)
Stage 4	0.889 (0.624, 0.971)	0.556 (0.305, 0.748)	0.444 (0.216, 0.651)	0.333 (0.137,0.545)	0.222 (0.069, 0.429)
**Survival by stage 1-5 years, not screened**					
Stage 1	0.967 (0.960, 0.973)	0.939 (0.930,0.947)	0.905 (0.895,0.915)	0.869 (0.856,0.880)	0.824 (0.810,0.837)
Stage 2	0.944 (0.937,0.950)	0.888 (0.879,0.896)	0.840 0.830,0.850	0.781 0.769,0.793	0.739 0.726,0.751
Stage 3	0.896 (0.887,0.905)	0.793 (0.780,0.805)	0.704 (0.690,0.718)	0.638 (0.623,0.652)	0.582 (0.566,0.597)
Stage 4	0.529 (0.510,0.547)	0.316 (0.299,0.333)	0.211 (0.196,0.226)	0.159 (0.145,0.172)	0.127 (0.115,0.139)

Source: Analysis of Victorian Cancer Registry dataset 2009-2019* under license for this study
