# Supplementary material for: Tailored recruitment interventions to improve bowel cancer screening in Arabic and Mandarin speaking groups: Modelled cost-effectiveness
Source: PLoS One. 2024 Nov 14;19(11):e0313058. doi: 10.1371/journal.pone.0313058 (PMC11563420; doi:10.1371/journal.pone.0313058)
Supplement: S3 Table — (PDF) [file pone.0313058.s003.pdf]

Table S3: Costs of Arabic and Mandarin program 2019

Category	Value	Details of cost
**Costs of Arabic recruitment interventions**		
Grant for One to one support sessions*, Organisation 1*	$7,000	Recruitment and communication of 118 community members, one-to-one sessions with 33 community members. 160 hours Arabic Welfare Facilitator's time – $35/hour, administrative costs- $765
Grant for Community Event, *Organisation 2*	$6,000	Community event and 10 sessions delivered to 543 people. Six Cancer Council Victoria staff members and two keynote speakers. Six staff time- $35/hour for a total of 125 hours, venue hire $600, administrative costs - $280, two keynote gift vouchers- $200.
Grant for Bowel cancer screening information sessions- facilitator and dietician, *Organisation 3*	$2,500	Recruitment of 57 men and women from Arabic speaking community. Two Cancer Council Victoria staff members- $35 x 55 hours, administration $350.
Arabic Stakeholders event	$722	Campaign launch with Arabic community and local media, filming community champions video
Arabic GP education dinner/event	$1,400	Cancer Council Victoria staff presented the importance of bowel cancer screening to GPs.
Playing cards	$1,248	100 playing cards in Arabic
Video editing	$2,860	Edited existing videos for social media and community education.
Radio ads Arabic	$2,520	Arabic radio ads: 42 slots across 3 stations. Played a bonus 12 times at no extra cost
Print media ads	$3,199	Half page ads in two Arabic newspapers
**Costs of Mandarin recruitment interventions**		
Grant for community education *Chinese Organisation*	$7,900	Funding provided to community agency to share video with their community stakeholders and conduct a radio interview to create awareness and deliver 19 sessions to a total of 470 community members.
Grant for GP education	$3,850	Agency was funded to organise a bowel cancer screening workshop for GPs serving the Chinese community. Cancer Council Victoria staff presented at this workshop that was attended by over 50 GPs (online)
Video edit	$143	Edited existing video for social media
Radio ads	$4,900	70 slots on one station. Ad received 35 bonus plays at no extra cost.
WeChat messaging	$5,000	Approx. 250,000 impressions
Newspaper ad	$4,800	Half page in 2 Chinese newspapers
**Costs incurred by both Arabic and Mandarin***		
In language brochures	$1,140	Printing 3000 x $0.38, Design & Translation $979
Education. materials	$1,780	Design and translation
Tote bags	$2,415	500 x $4.83
Social media production costs	$902	Graphic design
Facebook Video ads	$2,620	Targeted Facebook ads for 3 months
Cancer Council Victoria Staff time and overheads	$85,448	Community Engagement Officer 0.5 EFT, Community Engagement Coordinator 0.3 EFT, Workforce Engagement Coordinator 0.3 EFT, Media communications advisor 0.1 EFT

Notes: *Costs incurred by both Arabic and Mandarin groups are the total costs divided equally between the two groups.
