# Supplementary material for: Tailored recruitment interventions to improve bowel cancer screening in Arabic and Mandarin speaking groups: Modelled cost-effectiveness
Source: PLoS One. 2024 Nov 14;19(11):e0313058. doi: 10.1371/journal.pone.0313058 (PMC11563420; doi:10.1371/journal.pone.0313058)
Supplement: S4 Table — (PDF) [file pone.0313058.s004.pdf]

S4 Table: Results of varying discount rates.

**Arabic Program 0% discount**			
	**Base case (no targeted program)**	**0.2% increase in screening, 1 year**	**1.3% increase in screening, 1 year**
Mean QALYs gained per person	20.168 (20.137 to 20.188)	20.177 (20.146 to 20.196)	20.196 (20.166 to 20.214)
Mean Health-care costs per person	$1975 ($1374 to $2641)	$1992 ($1398 to $2658)	$1994 ($1393 to $2677)
Incremental cost-effectiveness ratio*	–	$2152 ($1241 to $2514)	$711 ($359 to $1062)
**Arabic Program 3.5% discount**			
Mean QALYs gained per person	12.900 (12.886 to 12.908)	12.906 (12.892 to 12.914)	12.919 (12.907 to 12.928)
Mean Health-care costs per person	$1042 ($771 to $1340)	$1056 ($790 to $1353)	$1056 ($786 to $1357)
Incremental cost-effectiveness ratio*	–	$2536 ($1842 to $3010)	$723 ($508 to $909)
**Mandarin Program 0% discount**			
	**Base case (no targeted program)**	**1.1% increase in screening, 1 year**	**2.4% increase in screening, 1 year**
Mean QALYs gained per person+	20.412 (20.381 to 20.432)	20.415 (20.384 to 20.437)	20.425 (20.393 to 20.447)
Mean Health-care costs per person	$1853 ($1219 to $2572)	$1851 ($1219 to $2567)	$1863 ($1238 to $2577)
Incremental cost-effectiveness ratio*	–	dominant (dominant to $5152)	$755 ($78 to $1739)
**Mandarin Program 3.5% discount**			
Mean QALYs gained per person	13.097 (13.085 to 13.105)	13.106 (13.094 to 13.114)	13.120 (13.107 to 13.128)
Mean Health-care costs per person	$924 ($641 to $1258)	$932 ($650 to $1263)	$944 ($666 to 1274)
Incremental cost-effectiveness ratio*	–	$863 ($497 to $1213)	$849 ($632 to $1155)

Notes: QALY: Quality adjusted life years
